# Design-related bias in studies investigating diagnostic tests for venous thromboembolic diseases: a systematic review and meta-analysis

**DOI:** 10.3389/fcvm.2024.1420000

**Published:** 2024-11-29

**Authors:** Laura Boschetti, Henning Nilius, Hugo Ten Cate, Walter A. Wuillemin, Livia Faes, Patrick M. Bossuyt, Lucas M. Bachmann, Michael Nagler

**Affiliations:** ^1^ Department of Clinical Chemistry, Inselspital University Hospital, University of Bern, Bern, Switzerland; ^2^ Division of Haematology, Cantonal Hospital of Lucerne, Lucerne, Switzerland; ^3^ Graduate School for Health Sciences, University of Bern, Bern, Switzerland; ^4^ Laboratory of Clinical Thrombosis and Haemostasis, and Cardiovascular Research Institute, Maastricht University Medical Center, Maastricht, Netherlands; ^5^ University of Bern, Bern, Switzerland; ^6^ Medignition Inc., Zurich, Switzerland; ^7^ Medical Faculty, University of Zurich, Zurich, Switzerland; ^8^ NIHR Biomedical Research Center, Moorfields Eye Hospital NHS Foundation Trust and UCL Institute of Ophthalmology, London, United Kingdom; ^9^ Department of Clinical Epidemiology, Biostatistics & Bioinformatics, Academic Medical Center-University of Amsterdam, Amsterdam, Netherlands

**Keywords:** diagnostic tests, sensitivity and specificity, meta-analysis, venous thromboembolism, venous thrombosis

## Abstract

**Background:**

Early detection and diagnosis of venous thromboembolism are vital for effective treatment. To what extent methodological shortcomings exist in studies of diagnostic tests and whether this affects published test performance is unknown.

**Objectives:**

We aimed to assess the methodological quality of studies evaluating diagnostic tests for venous thromboembolic diseases and quantify the direction and impact of design characteristics on diagnostic performance.

**Methods:**

We conducted a literature search using Medline and Embase databases for systematic reviews summarizing diagnostic accuracy studies for five target disorders associated with venous thromboembolism. The following data were extracted for each primary study: methodological characteristics, the risk of bias scored by the QUADAS QUADAS-2 instrument, and numbers of true-positives, true-negatives, false-positives, and false-negatives. In a meta-analysis, we compared diagnostic accuracy measures from studies unlikely to be biased with those likely to be biased.

**Results:**

Eighty-five systematic reviews comprising 1’818 primary studies were included. Adequate quality assessment tools were used in 43 systematic reviews only (51%). The risk of bias was estimated to be low for all items in 23% of the primary studies. A high or unclear risk of bias in particular domains of the QUADAS/QUADAS-2 tool was associated with marked differences in the reported sensitivity and specificity.

**Conclusions:**

Significant limitations in the methodological quality of studies assessing diagnostic tests for venous thromboembolic disorders exist, and studies at risk of bias are unlikely to report valid estimates of test performance. Established guidelines for evaluation of diagnostic tests should be more systematically adopted.

**Systematic Review Registration:**

PROSPERO (CRD 42021264912).

## Introduction

Treatment of venous thromboembolism can only be initiated once a diagnosis is established. The diagnostic workup is the first step in any medical encounter, and it is acknowledged that the quality of the diagnostic process determines the quality of care to a large amount (1). A delayed diagnosis of venous thromboembolic diseases such as pulmonary embolism or heparin-induced thrombocytopenia might result in severe damage, persistent sequelae, or even death (2, 3). Accordingly, false diagnosis and overtreatment might not only result in increased costs but also direct adverse events, the initiation of additional investigations, and withdrawal of treatments necessary for other diseases (4, 5). Key parts of the diagnostic work-up are medical tests such as laboratory assays or imaging studies (6). Thus, the effectiveness of the diagnostic process depends on the performance of respective tests (3). Suboptimal tests might lead to increased numbers of wrong diagnoses or unnecessary delays in securing a correct diagnosis (3, 4, 7). In addition, it has been recognized that sophisticated and expensive tests that are disseminated without suitable evaluations can have marginal clinical value and economic adversity (5).

Diagnostic accuracy studies evaluating the clinical performance of laboratory tests are an essential part of the implementation process (5). Using the diagnostic accuracy measures obtained in these studies, the pos*t*-test probability of a particular disease can be estimated in individual patients, thus clarifying the diagnostic utility of the test (8). However, methodological shortcomings and design-related bias of diagnostic accuracy studies can easily lead to biased study results and erroneous conclusions on the clinical value of medical tests (1). Well known historical examples and some empirical evidence illustrate how methodological shortcomings in diagnostic accuracy studies may lead to wrong conclusions and an unjustified subsequent implementation in clinical practice (9–14). A number of guidelines and tools for assessing and improving quality of studies evaluating diagnostic tests have been developed in the last decades to overcome these problems. In particular, the STARD guideline focuses on the accurate design and reporting of diagnostic accuracy studies and the QUADAS-2 tool assesses the methodological quality of studies to be used in systematic reviews and meta-analyses (15–17). From previous studies we know that non-adherence can generate bias (9–14). The question appears to what extent design-related deficiencies exist in studies of diagnostic tests for venous thromboembolism and whether these affect published test performance.

In this study we systematically assessed the methodological quality of studies evaluating diagnostic tests for venous thromboembolic diseases and quantified the direction and impact of studies at risk of bias on diagnostic performance.

## Methods

### Study design, search strategy, and data sources

A protocol was developed and submitted to the PROSPERO international register of systematic reviews (CRD 42021264912). A sensitive search strategy was developed to identify systematic reviews summarizing diagnostic accuracy studies for the tests used to diagnose venous thromboembolic diseases; the search strategy is given in the Supplementary Material. To get a comprehensive dataset, we included all disease entities in the search strategy that are associated with venous thromboembolism: venous thrombosis, pulmonary embolism, lower extremity deep vein thrombosis, heparin-induced thrombocytopenia, and disseminated intravascular coagulation. We decided against antiphospholipid antibody syndrome because it is often associated with other clinical sequalae. The search strategy was tested in a set of 10 index publications. The MEDLINE and EMBASE databases were searched without any restrictions regarding date or language. The database search was complemented by screening reference lists of included studies. The literature search was updated last time on the 20th of November 2020. The manuscript was prepared using the Preferred Reporting Items for Systematic Reviews and Meta-analysis (PRISMA) (18).

### Study eligibility

The literature search and selection of publications for full-text review was done by three authors (LB, TMR, LF). Full-text review and assessment for eligibility was done independently by two authors (LB, LF). Two reviewers (MN, LMB) arbitrated unclear cases. Inclusion criteria were (1) systematic review of diagnostic accuracy studies, (2) evaluating one or more index tests used to identify one of the mentioned disease entities, and (4) sufficient details from included studies to generate 2 by 2 contingency tables.

### Data extraction

All data were extracted in a standardized extraction form. The following data items were extracted from each included systematic review: number of studies, employment, and type of a quality assessment tool. The primary studies included in the systematic review were assessed to extract the following data: sample size, numbers of affected and unaffected patients, true positives, false positives, false negatives, and true negatives. In addition, the QUADAS QUADAS-2 rating assigned to these studies by the authors of the systematic review were recorded: risk of bias and applicability concerns with regard to (a) patient selection, (b) index test, (c) reference standard test, and (d) flow and timing. Four reviewers (LB, MN, LF, LMB) independently did data extraction and disagreement was resolved by consensus.

### Measures of methodological quality

We assessed the methodological quality of included primary studies in terms of precision and risk of bias. We first analyzed included primary studies in terms of sample size (19). We then evaluated whether an included systematic review had adequately applied a quality assessment tool. The methodological quality of the primary studies, as assessed by the review authors, was extracted, expressed as QUADAS/QUADAS-2 results. We decided to rely on the assessment of previous authors since the application of QUADAS-2 has to be done in the context of and adapted to the respective research question.

### Statistical analysis

Index tests were categorized into (a) ultrasound techniques, (b) other imaging studies, (c) laboratory tests, and (d) other tests. To assess the effects of methodological deficiencies on the reported diagnostic test accuracy, various meta-regression analyses were performed. Bivariate models as described by Reitsma et al. and implemented in the “mada” package for “R” were fitted to the data of each test category (20–22). We decided against performing a meta-analysis for the studies that were categorized as “other tests” because this group only contained 6 studies and estimates would be unstable. The bivariate Reitsma model is a random-effects linear mixed model that pools the logit transformed sensitivities and false-positive rates together.

Each of the QUADAS-criteria (coded as “low risk of bias” or “not low risk of bias”) was separately entered into the meta-regression as the independent variable. The back-transformed regression coefficients and corresponding 95% confidence intervals (CI) were then displayed in a forest plot using the “forestplot” package (23). The same procedure was followed to also analyze the impact of adjudication as the reference standard. For this analysis, we assumed that the heterogeneity between studies is high since they comprise different tests and, therefore, a random-effects model was chosen. For the purposes of the primary analysis, the effects of methodological deficiencies were assumed to be similar across test categories. As a a sensitivity analysis, we conducted a multivariable meta-regression analysis of the diagnostic odds ratios (DOR) using the “meta” and “metafor” packages for R. This analysis included adjustments for the meta-analysis and all domains of the QUADAS-2 tool. A forest plot showing the relative diagnostic odds ratios comparing low risk of bias high applicability with not low risk of bias not high applicability was created.

## Results

### Study selection

**Figure 1:**
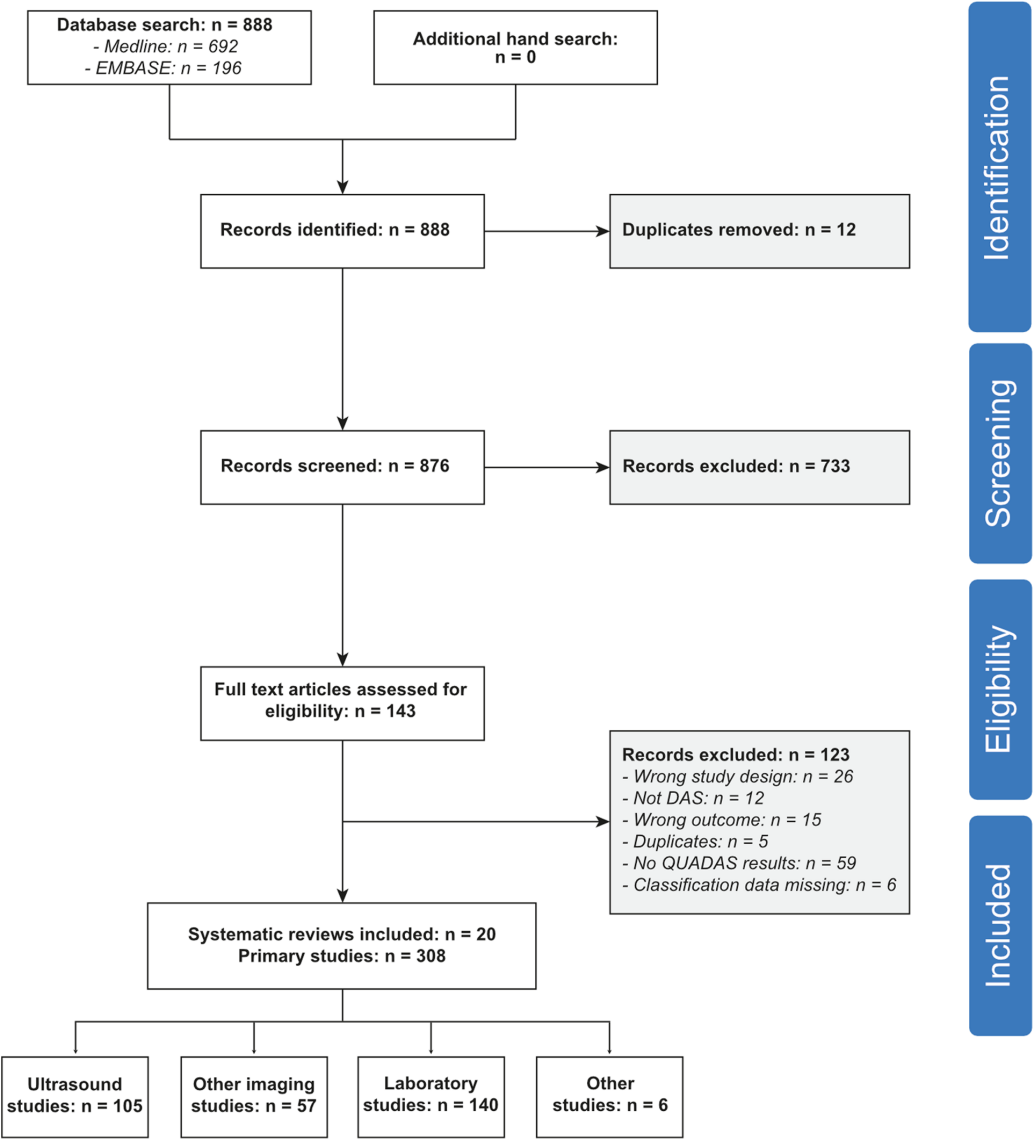
Flow of the studies.

After deduplication, we identified 876 potentially eligible articles (Figure 1). One-hundred and forty-three were selected for full-text screening. Out of these, we excluded 26 articles because of the publication type (no systematic review), 12 records because of a different scope, 15 articles because of insufficient data, and five more duplicates. Finally, we included 85 systematic reviews (8, 24–105), summarizing the results of 1'818 primary studies (Supplementary Table S1). For 308 primary studies, QUADAS/QUADAS-2 scores were available in 20 systematic reviews (8, 30, 31, 38, 46, 48–51, 53–55, 58, 67, 76, 81, 85, 93, 99, 104). These studies were included in our meta-analysis of design-related bias (Supplementary Table S2).

### Study characteristics and sample size

The characteristics of included systematic reviews are given in Supplementary Table S1. The included studies covered the whole spectrum of diagnostic problems associated with venous thromboembolism, and a wide range of diagnostic tests. The number of primary studies ranged from 3 to 108. QUADAS QUADAS-2 was used as a quality assessment tool in 43 systematic reviews (51%), and a self-constructed tool in 14 studies (16%). No formal quality assessment was done in 28 studies (33%). The systematic reviews were published between 1991 and 2020 (median 2014).

The characteristics of all primary studies included in the meta-analysis are reported in Supplementary Table S2. The number of patients ranged from 7 to 376 (median 159). The prevalence varied between 0% and 94% (median 23%). Laboratory tests were studied in 140 primary studies (46%), ultrasound techniques in 105 studies (34%), other imaging studies in 57 (18%), and other tests in 6 studies (2%).

### Quality assessment

Among 308 primary studies with QUADAS QUADAS-2 ratings available, a low risk of bias in all domains was reported in 120 studies only (39%; Supplementary Table S2). The risk of bias was estimated to be “high” or “unclear” with regard to the patient selection in 101 studies (36%), the index test in 82 studies (28%), the reference test in 102 primary studies (35%), and the flow and timing in 104 studies (36%). Applicability concerns were high or unclear with regard to patient selection in 56 studies (30%), with regard to index test in 28 studies (16%), and with regard to the reference standard test in 30 studies (16%). No applicability concerns in all domains were reported in 126 studies (62%).

### Design-related bias and published test performance

**Figure 2:**
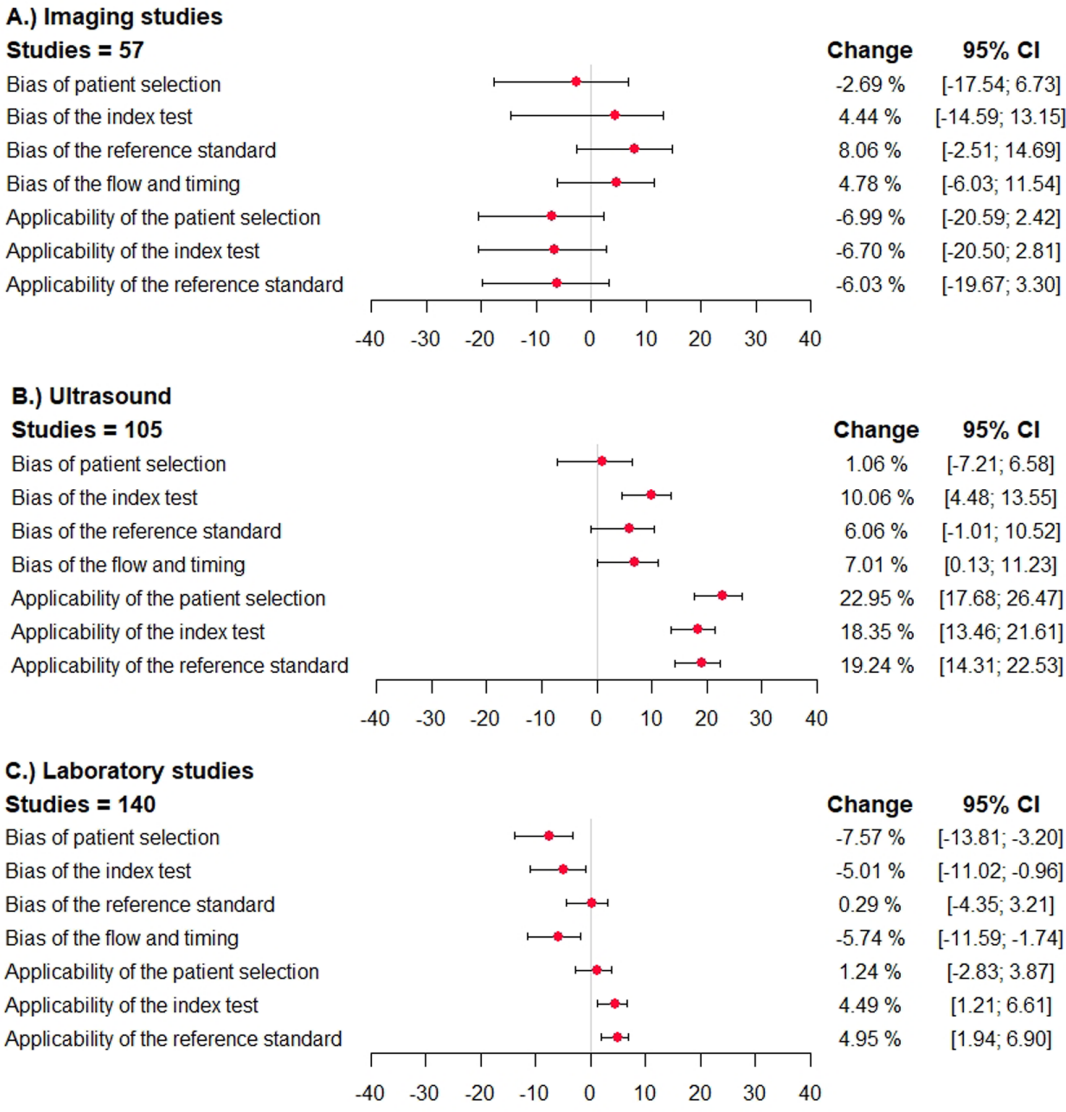
Direction and impact of methodological bias on summary sensitivity in diagnostic accuracy studies for venous thromboembolism. The difference in sensitivity and the 95% confidence interval is shown (percentages) in studies reporting on ultrasound studies, other imaging studies, and laboratory tests. The different domains of the QUADAS/QUADAS-2 tool are given. A deviation to the right corresponds to an overestimation of sensitivity, and a deviation to the left corresponds to an underestimation. **(A)** imaging studies, **(B)** ultrasound studies, **(C)** laboratory studies.

The direction and impact of methodological shortcomings on summary sensitivity are shown in Figure 2. The difference is shown per item of the QUADAS/QUADAS-2 tool in three index test categories (imaging studies, ultrasound studies, and laboratory studies).

**Figure 3:**
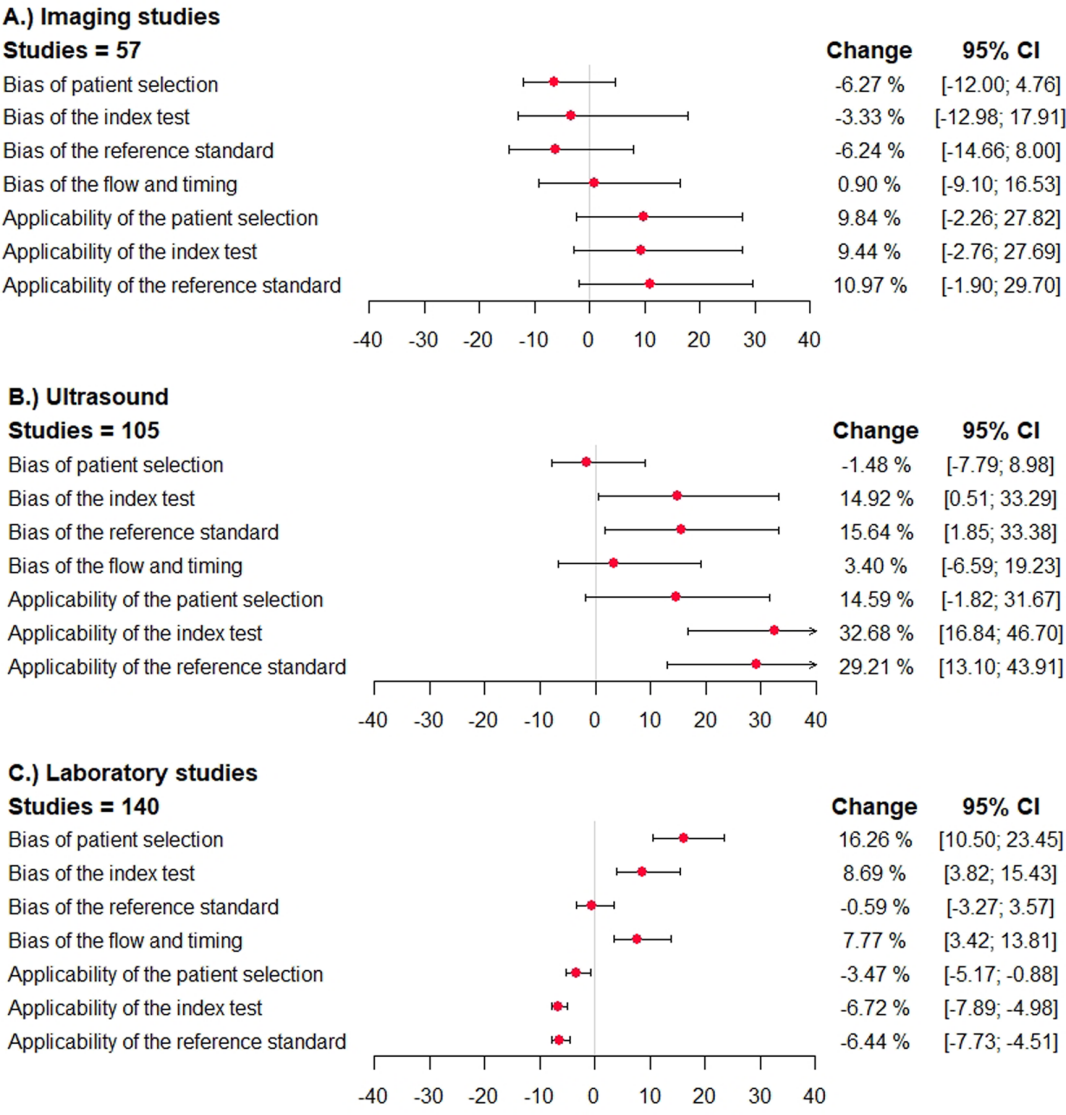
Direction and impact of methodological bias on summary specificity in diagnostic accuracy studies for venous thromboembolism. The difference in specificity and the 95% confidence interval is shown (percentages) in studies reporting on ultrasound studies, other imaging studies, and laboratory tests. The different domains of the QUADAS/QUADAS-2 tool are given. A deviation to the right corresponds to an overestimation of specificity, and a deviation to the left corresponds to an underestimation. **(A)** imaging studies, **(B)** ultrasound studies, **(C)** laboratory studies.

In primary studies categorized to have a high (or unclear) risk of bias with regard to *patient selection*, the summary sensitivity was lower in case of imaging studies [−2.7%; 95% confidence interval (−17.5; 6.7)], simila in case of ultrasound studies [1.1%; 95% confidence interval (−7.2; 6.6)], and significantly lower in laboratory tests [−7.6%; 95% confidence interval (−13.8; −3.2)]. The specificity was similar in case of imaging studies and ultrasound studies and significantly higher in studies assessing laboratory tests [16.3%; 95% confidence interval (10.5; 23.5)] (Figure 3).

In studies categorized to have a high risk of bias with regard to the *index test*, *reference standard test*, or *flow and timing*, the sensitivity was higher in case of imaging studies, ultrasound studies, and lower in laboratory tests (Figure 2). The specificity was mostly lower in imaging studies, and higher in ultrasound studies and laboratory tests (Figure 3).

In studies categorized to have applicability concerns, the sensitivity was lower in imaging studies, higher in case of ultrasound studies and laboratory studies. The specificity was higher in imaging studies and ultrasound studies, and significantly lower in laboratory tests.

**Figure 4:**
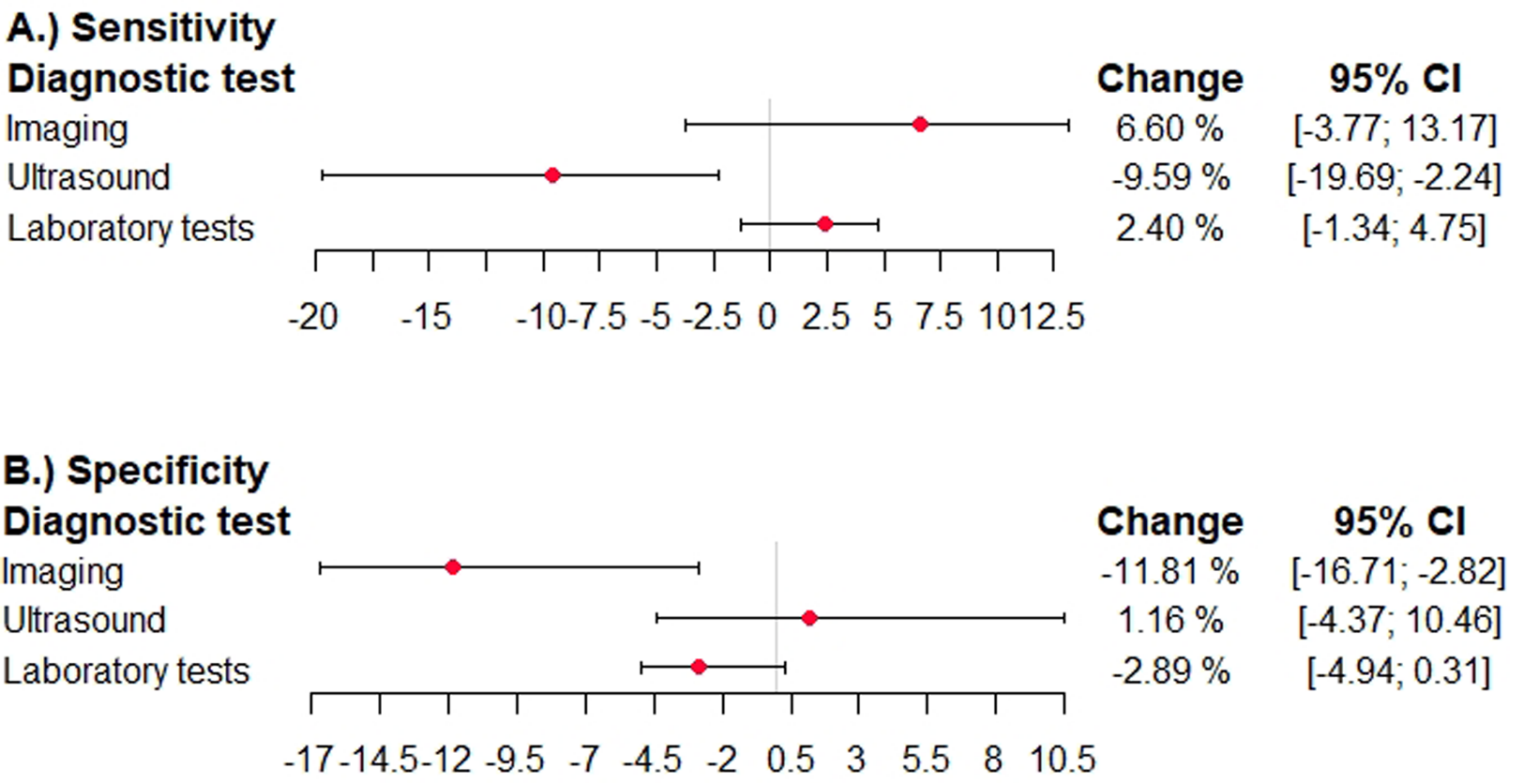
Effects of expert adjudication as reference standard on summary test performance in diagnostic accuracy studies for venous thromboembolism. The difference in sensitivity and specficitiy and the 95% confidence interval is shown in studies reporting on ultrasound studies, other imaging studies, and laboratory tests. The different domains of the QUADAS QUADAS-2 tool are given. A deviation to the right corresponds to an overestimation, and a deviation to the left corresponds to an underestimation. **(A)** sensitivity diagnostic test, **(B)** specificity diagnostic test.

In studies using adjudication as the reference standard, the sensitivity was higher in imaging tests, significantly lower in ultrasound studies [−9.6%; 95% confidence interval (−19.7; −2.2)], and higher in laboratory tests (Figure 4). The specificity was significantly lower in imaging tests [−11.8%; 95% confidence interval (−16.7; −2.8)], higher in ultrasound studies and lower in laboratory tests (Figure 4).

### Sensitivity analysis

As a sensitivity analysis we performed a multivariable meta regression using DOR. When adjusting for the meta-analysis effects were attenuated and did not achieve statistical significance. Reported DOR were higher in studies with non-low risk of bias in the domains of the index test (RDOR 1.45; 95% CI 0.75, 2.84), reference standard (RDOR 1.22; 95% CI 0.62, 2.41), flow and timing (RDOR 1.21; 95% CI 0.72, 2.22) and non-high applicability of the reference standard (RDOR 1.94, 95% CI 0.72, 5.26). A lower reported DOR was present in studies with non-low risk of bias in the patient selection (RDOR 0.74; 95% CI 0.36, 1.51) and non-high applicability in the patient selection (RDOR 0.88; 95% CI 0.37, 2.09) and in the performance of the index test (RDOR 0.54; 95% CI 0.20, 1.46).

## Discussion

In a comprehensive systematic review, we found significant shortcomings in the methodological quality of studies included in 85 systematic reviews of diagnostic tests used to diagnose venous thromboembolic disorders. Adequate quality assessment instruments were used in half of the systematic reviews only. Among 308 primary studies included in a meta-analysis, the number of patients was limited and a low risk of bias in all domains was reported in 120 studies only (39%). A high or unclear risk of bias in particular domains of the QUADAS/QUADAS-2 tool was associated with marked differences in the reported sensitivity and specificity.

We are unaware of previous studies investigating extent and effects of methodological shortcomings systematically in diagnostic accuracy studies for venous thromboembolic diseases. Our results are in-line with previous publications in general medical journals confirming that methodological shortcomings are common and the quality of reporting restricted (9, 12). Some evidence exists regarding a systematic bias due to methodological shortcomings. In 1999 Lijmer and colleagues reported on systematic overestimation of the diagnostic performance of a test in studies in which particular methodological requirements were not met (9). The issues they found to have a high risk correspond very well to the domains of the QUADAS-2 tool we identified as such: “patient selection” and “reference test”. Rutjes et al. also focused on a number of methodological factors associated with a risk of bias in diagnostic accuracy estimates (12). In agreement with our results, these authors identified issues associated with patient selection as particularly sensitive to bias. Our results are also in line with a systematic review and meta-analysis of studies investigating the accuracy of magnetic resonance imaging in detecting silicone breast implant ruptures (11). The authors identified patient selection procedure as particularly sensitive to bias. Other authors mentioned disease prevalence and details of data analysis as potential sources of bias (106). In contrast to these previous investigations, we observed a relationship between bias and quality status according to QUADAS-2. Boyer and colleagues studied diagnostic accuracy studies of carpal tunnel syndrome and concluded that these studies are unlikely to report results that are applicable to actual clinical practice (107). Fontela and colleagues found that quality and reporting was limited in diagnostic accuracy studies focused on TB, HIV and Malaria (108). Other studies in other domains found that the sample size in diagnostic accuracy studies was limited and *a priori* sample size calculations were rare (19, 109).

In this investigation, we studied methodological issues in a large number of studies investigating a broad range of diagnostic tests for detecting all important diseases associated with venous thromboembolism. Arguably our sample is a positive selection of the full body of evidence, since only studies included in systematic reviews were considered. Furthermore, there could also be a publication bias, since studies with negative results are less frequently published. Thus, the actual problem might be worse when considering the complete diagnostic literature on venous thromboembolic diseases. As another important limitation, we are not able to conduct sensitivity analyses with single diagnostic tests because of the numbers of diagnostic accuracy studies available. This reflects the general methodological problems and limitations in the validity of previous studies. The risk of bias is estimated by higher or lower measures of accuracy in the presence or absence of certain methodological limitations. In every analysis, a group of diagnostic tests is analyzed that is more or less heterogeneous. This introduces a potential confounder whose influence can hardly be accounted for. To directly measure the effect of methodological aspects on reported diagnostic accuracy, and thus the extent of bias, would require a large number of studies of different designs of the same diagnostic test. As these do not yet exist, the bias cannot be measured directly. As a further limitation, we chose a certain set of diseases with VTE, and we decided against the antiphospholipid antibody syndrome (because it often has other clinical sequelae). We cannot completely rule out the possibility that the results of our study would have been different. Another limitation is that we cannot go back to the level of individual tests and indicate the extent of over- or underestimation. The main reason for this is that we do not know the “true” value. Another reason is that there are too few studies within each test and therefore too little variance in the quality variables. However, we have now uploaded the raw data as Excel files in the Supplementary Material. Interested readers can go back to the study or test level and look at the methodological quality and the reported diagnostic utility.

Using a comprehensive approach, we obtained empirical evidence for significant shortcomings in the methodological quality of studies assessing diagnostic tests for venous thromboembolic diseases. Although deviations can take on very different directions and extents depending on the type of methodological limitation and diagnostic test, aspects of applicability are significant. Interestingly, laboratory tests appear particularly prone to deviations. Moreover, the results of our meta-analysis suggest that these shortcomings can result in biased accuracy estimates. This observation calls for increased efforts to implement current guidelines for reporting and assessment of methodological quality (15, 17)*.* Several authors have demanded a phased evaluation of medical tests, in parallel with the evaluation required for FDA-approval of new drugs (5, 7, 110–112). In a first phase, the analytical characteristics and the technical accuracy including reproducibility are evaluated. In a second phase, the diagnostic accuracy will be investigated in an adequate study design. These studies will be complemented by a phase three determining health outcomes (mortality and morbidity) of using the test. Afterwards, the cost-effectiveness must be evaluated, decision-making algorithms developed, and the organizational impact evaluated. The advantage of a phased evaluation is that further evaluation will be stopped after insufficient results at an early stage. Thus, significant harm to patients associated with a premature implementation of medical tests will be avoided. Furthermore, a relevant amount of costs that are associated with the use of tests with unclear value can be saved.

This comprehensive systematic review identified significant limitations in the methodological quality of studies assessing diagnostic tests used to diagnose venous thromboembolic disorders. Design-related shortcomings were associated with marked differences in the reported sensitivity and specificity. Our data suggest that studies at risk of bias because of methodological shortcomings are unlikely to report valid estimates of test performance.

## Data Availability

The raw data supporting the conclusions of this article will be made available by the authors, without undue reservation.
