# Supplementary material for: Design-related bias in studies investigating diagnostic tests for venous thromboembolic diseases: a systematic review and meta-analysis
Source: Front Cardiovasc Med. 2024 Nov 29;11:1420000. doi: 10.3389/fcvm.2024.1420000 (PMC11638182; doi:10.3389/fcvm.2024.1420000)
Supplement: Supplementary file 1 [file Datasheet1.pdf]

Supplementary Material

1. Contents:

1 Supplementary Data

1.1 Search strategy

2 Supplementary Figures and Tables

2.1 Supplementary Table 1 (separate excel file): Characteristics of systematic reviews summarizing diagnostic accuracy studies for tests used to diagnose five target disorders associated with venous thromboembolism.

2.2 Supplementary Table 2 (separate excel file): Characteristics of primary studies included in meta-analysis of design-related bias. All data retrieved are given.

2.3 Supplementary Table 3: List of abbreviations

1 Supplementary Data

1.3 Search strategy 20.11.2020

2. PubMed

3. (("Thrombocytopenia"[MeSH Terms] AND "Heparin"[MeSH Terms]) OR "Pulmonary Embolism"[MeSH Terms] OR "Venous Thromboembolism"[MeSH Terms] OR ("Lower Extremity"[MeSH Terms] AND "Venous Thrombosis"[MeSH Terms]) OR "Disseminated Intravascular Coagulation"[MeSH Terms] OR "Heparin-induced thrombocytopenia"[Title/Abstract] OR "lung embolism*"[Title/Abstract] OR "pulmonary embolism*"[Title/Abstract] OR "Deep venous thrombosis"[Title/Abstract] OR "deep vein thrombosis"[Title/Abstract] OR "DVT"[Title/Abstract] OR "Thrombophlebitis"[Title/Abstract] OR (("lower limb*"[Title/Abstract] OR "lower extremit*"[Title/Abstract]) AND "thrombosis"[Title/Abstract]) OR "LLDVT"[Title/Abstract] OR "LEDVT"[Title/Abstract] OR "disseminated intravascular coagulation*"[Title/Abstract] OR "disseminated intravascular coagulopath*"[Title/Abstract] OR "DIC"[Title/Abstract]) AND ("Sensitivity and specificity"[MeSH Terms] OR "predict*"[Text Word] OR "diagnos*"[Text Word] OR "accura*"[Text Word]) AND ("meta analysis"[Title/Abstract] OR "systematic review"[Title/Abstract])

4. Embase

5. ('meta analysis':ab,ti OR 'systematic review':ab,ti) AND (sensitiv*:ab,ti OR 'diagnostic accuracy'/de OR diagnostic:ab,ti) AND ('heparin induced thrombocytopenia'/exp OR 'lung embolism'/exp OR 'venous thromboembolism'/exp OR 'venous thromboembolism':ti,ab OR 'lower extremity deep vein thrombosis'/exp OR 'disseminated intravascular clotting'/exp OR 'heparin induced thrombocytopenia':ti,ab OR 'lung embolism*':ti,ab OR 'pulmonary embolism*':ti,ab OR 'deep venous thrombosis':ti,ab OR 'deep vein thrombosis':ti,ab OR dvt:ti,ab OR thrombophlebitis:ti,ab OR (('lower limb*':ti,ab OR 'lower extremit*':ti,ab) AND thrombosis:ti,ab) OR lldvt:ti,ab OR ledvt:ti,ab OR 'disseminated intravascular coagulopath*':ti,ab OR 'disseminated intravascular coagulation*':ti,ab OR DIC:ti,ab) AND [embase]/lim NOT ([embase]/lim AND [medline]/lim)

2 Supplementary Figures and Tables

2.3 Supplementary Table 3: List of abbreviations

**List of abbreviations**	
#	Numeric reference
AVDSf	alveolar dead space fraction
CLIA	Chemiluminescence Immunoassays
CT	computed tomography
CTPA	CT pulmonary angiography
CUS	compression ultrasound
cXR	Chest x-ray
DD	D-dimer
DVT	deep venous thrombosis
ELISA	enzyme-linked Immunosorbent Assay
EPPU	emergency physician-performed ultrasond
ETCO2	end-tidal carbon dioxide pressure
ETCO2/O2	end-tidal ratio of carbon dioxide to oxygen
FDG-PET	fluorodeoxyglucose (FDG)-positron emission tomography (PET)
HIPA	heparin-induced platelet activation test
HIT	heparin induced thrombocytopenia
IPG	impedance plethysmography
Latex	latex agglutination assay
LFI	lateral flow immunoassay
MCTPA	multi-detector computed tomography pulmonary angiography
MPV	mean platelet volume
MRI	magnetic resonance imaging
PA	pulmonary angiography
PaGIA	particle Gel Immuno Assay
PAT	heparin-induced platelet aggregation test
PCUS	point of care ultrasound
PE	pulmonary embolism
PERC	pulmonary embolism rule-out criteria
POCT	point of care testing
S-CTPA	spectral CT pulmonary angiography
sPsel	soluble P selectin
SRA	serotonin release assay
US	ultrasound

V/Q	ventilation perfusion scintigraphy
Vcap	volumetriccapnography
VCUS	lower extremity venous compression ultrasonography
VTE	venous thromboembolism
